# Successful treatment of systemic AL amyloidosis with autologous hematopoietic stem cell transplantation combined with cell‐free and concentrated ascites reinfusion therapy

**DOI:** 10.1002/ccr3.7233

**Published:** 2023-05-09

**Authors:** Satoko Oka, Kazuo Ono

**Affiliations:** ^1^ Division of Hematology Japanese Red Cross Society Wakayama Medical Center Wakayama Japan; ^2^ Division of Pathology Japanese Red Cross Society Wakayama Medical Center Wakayama Japan

**Keywords:** cardiac involvement, cell‐free and concentrated ascites reinfusion therapy (CART), hematopoietic stem cell transplantation (HSCT), immunoglobulin light chain (AL) amyloidosis

## Abstract

**Key Clinical Message:**

AL patients develop the unique toxicities of fluid retention and non‐cardiogenic pulmonary edema during the course of stem cell mobilization. We propose mobilization with CART as effective and safe treatment for AL patients with refractory anasarca.

**Abstract:**

We describe a 63‐year‐old male with systemic immunoglobulin light chain (AL) amyloidosis with cardiac, renal, and liver involvement. After four courses of CyBorD, mobilization with G‐CSF at 10 μg/kg was initiated and CART was simultaneously performed for fluid retention. No adverse events were observed during collection or reinfusion. Anasarca gradually disappeared and he underwent autologous hematopoietic stem cell transplantation. The complete remission of AL amyloidosis has been maintained, and the condition of the patient has remained stable for 7 years. We propose mobilization with CART as an effective and safe treatment option for AL patients with refractory anasarca.

## Graphical abstract

A 63‐year‐old male with systemic AL amyloidosis with cardiac, renal, and liver involvement. Computed tomography (CT) scan showing massive ascites at presentation (A, B). After 4 courses of CyBorD and mobilization with G‐CSF at 10 μg/kg was initiated. CT scan taken after G‐CSF for mobilization showing an increase in massive ascites (C, D). CART was simultaneously performed for fluid retention. CT scan taken after CART and autologous stem cell transplantation showing improvements (E, F).

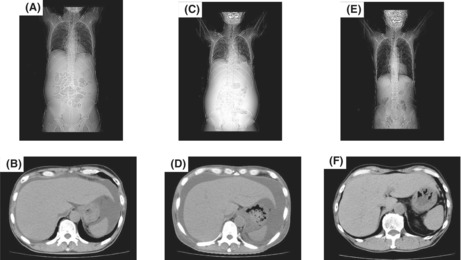

## 1 INTRODUCTION

Immunoglobulin light chain (AL) amyloidosis is a clonal plasma cell disorder that leads to progressive and life‐threatening organ failure. Although AL amyloidosis may involve any organ, the heart and kidneys are the most commonly affected organs. The presence and severity of cardiac disease is typically a powerful predictor of overall survival (OS).^1^ Other adverse predictors of survival include the involvement of two or more organs, an elevated level of β2‐microglobulin, and time to the referral center.^2^, ^3^ The median OS of patients with AL amyloidosis is approximately 1.1 years from the diagnosis of cardiac involvement and 0.75 years from the onset of heart failure.^4^ Median OS is shorter in patients with cardiac amyloidosis than in those without cardiac involvement.^5^ However, recent advances in treatments directed towards plasma cell dyscrasia have markedly prolonged OS.^6^ Chemotherapy and autologous hematopoietic stem cell transplantation (HSCT) have extended survival, with hematologic remission being documented. In a retrospective analysis of cyclophosphamide, dexamethasone, and bortezomib for AL patients, the condition of 13/17 patients improved and they subsequently underwent HSCT.^7^, ^8^ The hematologic response rate in this series of patients was higher than 90%.

However, AL patients develop fluid retention and non‐cardiogenic pulmonary edema during the course of stem cell mobilization, which may lead to a mortality rate of 2%–3% in the prechemotherapy phase.^9^, ^10^ Some patients cannot proceed beyond mobilization because of heart failure or cardiac dysrhythmia. Therefore, supportive management is needed, particularly for AL with cardiac involvement. Cell‐free and concentrated ascites reinfusion therapy (CART) is used to treat refractory ascites and is safe and effective for cancer and cirrhosis.^11^ However, limited information is currently available on the effects of CART in patients with HSCT.

We herein presented an AL patient with cardiac involvement who developed fluid retention during the course of stem cell mobilization that was controlled by CART. After autologous HSCT, the patient maintained complete remission (CR) for 7 years. We propose mobilization with CART as an effective and safe treatment option for patients with systemic AL and refractory ascites.

## 2 CASE REPORT

**FIGURE 1:**
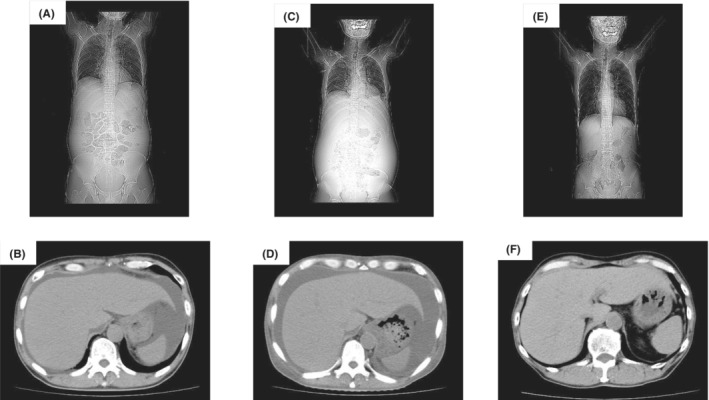
(A, B) Computed tomography (CT) scan showing massive ascites at presentation. (C, D) CT scan taken after G‐CSF for mobilization showing an increase in massive ascites. (E, F) CT scan taken after CART and autologous stem cell transplantation showing improvements.

**FIGURE 2:**
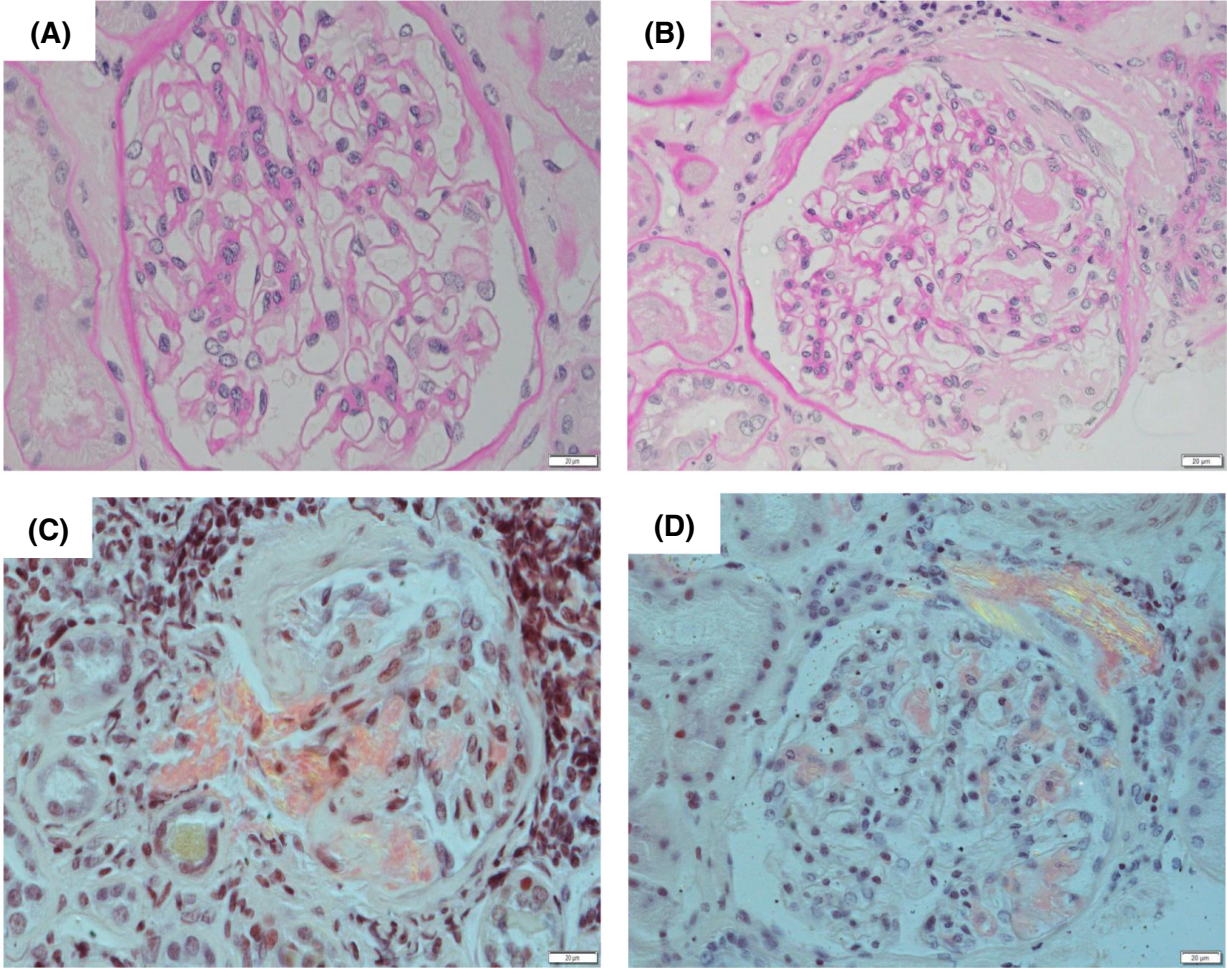
A histopathological analysis revealed non‐AA amyloid deposits in the kidney. (A, B) Renal biopsy samples indicated mesangial expansion with eosinophilic material (Periodic Acid‐Schiff staining, ×40). (C, D) Congo red staining revealed orange‐red deposits, which showed apple‐green birefringence under polarized light, in the vascular walls and glomeruli (×40).

**FIGURE 3:**
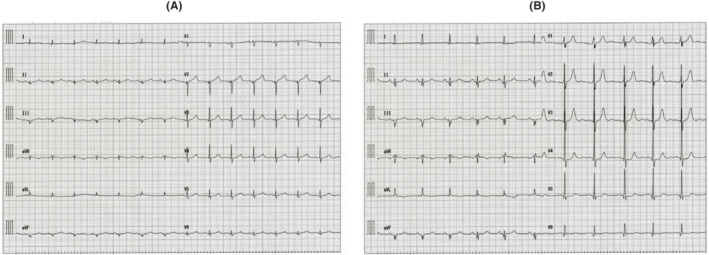
(A) Electrocardiography (ECG) at presentation showed a sinus rhythm with a low QRS voltage. (B) ECG normalized after autologous stem cell transplantation.

**FIGURE 4:**
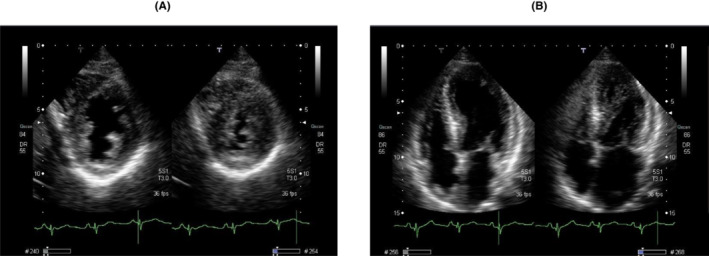
(A, B) Echocardiography showed a markedly increased left ventricular wall thickness with reduced contractions in posterior and lateral regions.

A 63‐year‐old male presented to our hospital with severe upper and lower edema and weight gain of 5 kg in the last 3 months (Figure 1A,B). A physical examination revealed blood pressure of 90/60 mmHg and ascites. Laboratory tests showed a white blood cell count of 72 × 10^9^/L, red blood cell count of 360 × 10^10^/L, hemoglobin concentration of 12.1 g/dL, and platelet count of 22.8 × 10^9^/L. His serum albumin level was 1.48 g/dL (normal range: 3.9–4.9 g/dL), alkaline phosphatase was 787 U/L (normal range: 104–338 U/L), β2 microglobulin was 3.1 μg/mL (normal range: <2 μg/mL), and estimated urinary total protein was 894 mg/dL (normal range: 0–20 mg/dL). The patient was diagnosed with nephrotic syndrome and kidney biopsy was performed. Light microscopy indicated mesangial expansion with eosinophilic material (Figure 2A,B). Congo red staining revealed orange‐red deposits, which showed apple‐green birefringence under polarized light in the vascular walls and glomeruli (Figure 2C,D). Serum protein electrophoresis and free light chain (FLC) assays showed an elevated λ‐FLC level of 224 mg/L (normal range: 5.7–26.3 mg/L) with a *κ*:*λ* ratio of 0.09 (normal range: 0.26–1.65). A bone marrow examination revealed 3% plasma cells. In laboratory tests, the level of N‐terminal brain natriuretic peptide (NT‐proBNP) was high (674 pg/mL [normal range: <125 pg/mL]) and that of troponin I was slightly elevated (312 ng/mL [normal range: <26.2 pg/mL]). A sinus rhythm with a low QRS voltage was observed on electrocardiography (ECG) (Figure 3A). Echocardiography showed a markedly increased left ventricular wall thickness with reduced contractions in the posterior and lateral regions (Figure 4A,B).

The patient was diagnosed with primary AL amyloidosis with cardiac, renal, and liver involvement. The patient received cyclophosphamide (200 mg on days 1, 8, 15, and 22, p.o.), bortezomib (1.3 mg/m^2^ on days 1, 4, 8, and 11 every 3 weeks, s.c.), and dexamethasone (40 mg/day on days 1–4 (all cycles) and 9–12 (cycles 1 and 2), p.o.) (CyBorD). After four courses of CyBorD, the patient achieved a hematologic complete response. Mobilization with G‐CSF at 10 μg/kg was initiated and fluid retention was detected (Figure 1C,D). CART was simultaneously performed for refractory ascites. An AHF‐MO ascitic filtration filter and AHF‐UP ascitic concentration filter (Asahi Kasei Medical) were used for CART. Ascites was processed at 2 L/h to prevent the development of fever. A total of 5.5 L of ascites fluid was collected in CART. No adverse events were observed during collection or reinfusion. Anasarca gradually disappeared and the volume of urine increased. The patient underwent harvesting when his WBC count was 10.0 × 10^9^/L. In July 2015, he was conditioned with melphalan (200 mg/m^2^), followed by the infusion of CD34 at a total dose of 1.9 × 10^6^/kg. His serum albumin level gradually increased to 3.3 g/dL, and the urinary protein creatinine ratio decreased from 4.38 to 1.23. His ALP level normalized (320 U/mL), and NT‐proBNP and troponin I levels both decreased (243 pg/mL and 23.3 ng/mL, respectively). ECG also normalized (Figure 3B). The condition of the patient has remained stable for 7 years with no further evidence of relapse (Figure 1E,F).

## 3 DISCUSSION

Cardiac involvement is a major factor affecting the survival of patients with AL amyloidosis. Untreated patients with cardiac amyloidosis have historically had a median survival of 6–12 months; however, the clinical use of molecularly targeted drugs, such as proteasome inhibitors and immunomodulatory agents, monoclonal antibodies such as daratumumab, have markedly prolonged OS.^7^ High dose chemotherapy with HSCT had a significant positive impact on AL patients who achieved a CR^8^; however, it is now less commonly used as initial therapy for patients with cardiac involvement because of high treatment‐related mortality.

Many patients with AL already have severe organ damage and, thus, supportive care forms the foundation of the success of HSCT in AL. AL patients develop the unique toxicities of fluid retention and non‐cardiogenic pulmonary edema during the course of stem cell mobilization that may lead to a mortality rate of 2%–3% in the prechemotherapy phase.^9^, ^10^ Some patients cannot proceed beyond mobilization because of heart failure or cardiac dysrhythmia. Management includes the close monitoring of weight during the administration of growth factor, salt restriction, the judicious use of diuretics, a lower dose and split schedule of granulocyte colony‐stimulating factor, and the avoidance of chemotherapy as part of the mobilization regimen, particularly for AL with cardiac involvement.

CART, a treatment system for ascites that was developed in 1977, removes unnecessary components in ascites using a filtration membrane and intravenously reinfuses collected proteins. CART is expected to improve quality of life and increase urine output by maintaining hemodynamics through increases in serum albumin levels. CART is safe and effective as a treatment option for large‐volume paracentesis with the infusion of albumin for cancer or cirrhosis.^12^ Limited information is currently available on the effects of CART in patients with HSCT,^13^, ^14^ and this is the first case report of mobilization with CART. The monitoring of body weight and the use of CART resulted in successful mobilization.

In the present case, fluid retention during the course of stem cell mobilization was controlled by CART and CR was achieved after PBSCT. After 7 years, the patient has remained in CR with the resolution of peripheral edema and a decrease in the level of NT‐proBNP. We propose mobilization with CART as an effective and safe treatment option for AL patients with refractory anasarca.

In conclusion, the early diagnosis of AL is important for the prevention of advanced damage and relapse while controlling plasma dyscrasia with highly effective treatments. The decision to treat this condition using aggressive measures with CART must not be delayed until advanced damage.

## AUTHOR CONTRIBUTIONS

**Satoko Oka:** Writing – original draft. **Kazuo Ono:** Writing – original draft.

## CONFLICT OF INTEREST STATEMENT

All authors have no conflict of interest.

## CONSENT

Written informed consent was obtained from the patient to publish this report in accordance with the journal's patient consent policy.

## Data Availability

All data generated or analyzed during this study are included in this published article.
